# Fusing autonomy and sociality via embodied emergence and development of behaviour and cognition from fetal period

**DOI:** 10.1098/rstb.2018.0031

**Published:** 2019-03-11

**Authors:** Yasuo Kuniyoshi

**Affiliations:** Next Generation Artificial Intelligence Research Center & School of Information Science and Technology, The University of Tokyo, 7-3-1 Hongo, Bunkyo-ku, Tokyo 113-8656, Japan

**Keywords:** human-centred AI/robotics, embodiment, emergent behaviour, development, autonomy, sociality

## Abstract

Human-centred AI/Robotics are quickly becoming important. Their core claim is that AI systems or robots must be designed and work for the benefits of humans with no harm or uneasiness. It essentially requires the realization of autonomy, sociality and their fusion at all levels of system organization, even beyond programming or pre-training. The biologically inspired core principle of such a system is described as the emergence and development of embodied behaviour and cognition. The importance of embodiment, emergence and continuous autonomous development is explained in the context of developmental robotics and dynamical systems view of human development. We present a hypothetical early developmental scenario that fills in the very beginning part of the comprehensive scenarios proposed in developmental robotics. Then our model and experiments on emergent embodied behaviour are presented. They consist of chaotic maps embedded in sensory–motor loops and coupled via embodiment. Behaviours that are consistent with embodiment and adaptive to environmental structure emerge within a few seconds without any external reward or learning. Next, our model and experiments on human fetal development are presented. A precise musculo-skeletal fetal body model is placed in a uterus model. Driven by spinal nonlinear oscillator circuits coupled together via embodiment, somatosensory signals are evoked and learned by a model of the cerebral cortex with 2.6 million neurons and 5.3 billion synapses. The model acquired cortical representations of self–body and multi-modal sensory integration. This work is important because it models very early autonomous development in realistic detailed human embodiment. Finally, discussions toward human-like cognition are presented including other important factors such as motivation, emotion, internal organs and genetic factors.

This article is part of the theme issue ‘From social brains to social robots: applying neurocognitive insights to human–robot interaction’.

## 1. Introduction

Human-centred AI/robotics [1–3] are rapidly becoming important as the real-world application of AI/robotics is boosted by recent technological advances. Their core claim, also shared with related/similar concepts such as beneficial AI [4,5], human-friendly robotics [6] and human–robot symbiosis [7,8], is that AI systems or robots must be designed and work for the benefits of humans with no harm or uneasiness.

In other words, the systems should be able to handle open-ended situations and tasks, reliably achieving effects in alignment with human values, safely and in a manner acceptable to humans. They should also be able to communicate with humans about self-behaviour/reasoning and human intention/values/emotion/feelings. And these capabilities must be unified and maintained even in unexpected circumstances beyond the range of pre-programming or pre-training. In short, achieving autonomy, sociality and their fusion [9] at any time under any circumstances is fundamentally important for intelligent systems in the real world.

It has been pointed out over the past three decades that a robust (i.e. effective in a wide range of situations) intelligent system for the real world cannot be built by combining independent information processing units [10] for recognition, action, decision-making, language, etc. This is mainly because the internal representations (i.e. symbols/numbers defined by the system designer to represent certain world/cognitive states and convey the input/output between the units) can mean different things to different units depending on various real-world situations, often leading to inconsistencies.

### (a) Embodiment

The early proposed solution [11,12] was to build a robot system as a collection of parallel independent units all directly interfaced to the world through perception and action, without internal representations. The units are effectively integrated by interacting with the environment (surrounding world relevant to the system) through a shared body, in the sense that the outputs drive the body and its affecting objects, complying with physics and geometric constraints, entailing an integrated effect as well as consistent changes on the sensor inputs that in turn change the outputs.

Such characteristics of the body–environment system are called *embodiment* [13]. It casts stable *constraints* on, and *shapes*, an open-ended interaction between the system and environment [14]. A simple example is when you swing your arm, its postural trajectories can take infinite variations but always constrained by the distances between the joints. The constraints are *orthogonal* to the system–environment interaction in the sense that they do not specify individual states/actions but impose a set of conditions/relations that the involved states/actions and their temporal derivatives must always satisfy.

Therefore, the embodiment is important in dealing with open-ended interactions including unexpected ones, because it does not specify or depend on individual input/output like supervised learning of convolutional neural networks (CNNs) widely used as core components of modern deep learning AI systems. Moreover, when the embodiment of a robot is somewhat common with humans, it provides ‘sensibility’ to its type of behaviour in the sense that it complies with the partial common constraints with humans and therefore cannot be totally bizarre, in just the same way that we regularly make sense of dog behaviour. And embodiment even ‘shapes the way we think’ [15]. This sets the ground for fusing autonomy and sociality.

### (b) Development

The above early solution works for low-level behaviours like those of insects but is not likely to scale up to higher cognition such as thoughts and communication that certainly requires internal representations. The *symbol grounding problem* [16] states the essential difficulty of always connecting such representations correctly to the real-world entities/events. However, researchers have pointed out that the problem may disappear if we look at the problem from the perspectives of natural evolution or ontogenetic development and have shown how representations and language can *emerge* from agent–environment/inter-agent interactions [17–20]. *Developmental robotics*, aka. *epigenetic robotics* or *autonomous mental development* [21–25], attempts to model ontogenetic development of human cognition and behaviour through robots accommodating knowledge from developmental sciences.

Some important developmental events, such as acquisition of behaviour imitation capability [26–29], object knowledge/affordances (knowing what actions can be done about it) [30–32], joint attention (attention shared with another) [33,34], concepts and language [19,20] have been modelled and demonstrated by robots. However, if we end up with independent learning models for different cognitive/behavioural functions, we will be stuck again with another integration problem similar to the one discussed at the beginning of this section. Therefore, it is very important to achieve continuous autonomous development [25,35,36] in which the *same* system acquires one function after another from the very bottom up to high cognition. If this is realized with the integration of details on individual developmental events as referenced above, it will solve all the problems discussed so far. It will start from trivial sensory–motor interaction via embodiment and gradually acquire new/higher cognitive/behavioural functions up to human-like cognition while the entire system is in action, always assuring the integrity, consistency and grounding to embodiment [37]: fusing autonomy and sociality.

### (c) From the beginning

A dynamical system view of human development [38] emphasizes continuous autonomous development as a change within a complex dynamical system, emerging from many interactions occurring in real time. A divide and conquer, or reductionist approach does not work for such a target. As discussed before, functional decomposition or temporal decomposition (into individual developmental events) should be avoided. An alternative approach is to begin with the following unanswered question: Where and how does it start? This is crucial for constructing a single system capable of continuous autonomous development, as discussed above.

**Figure 1.**
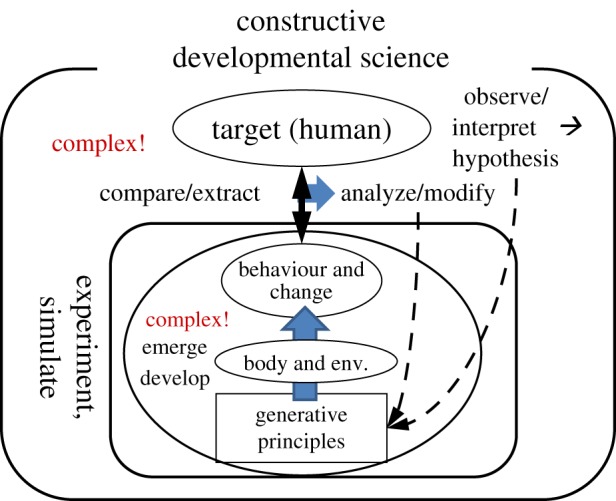
Constructive developmental approach. Generative principles are hypothesized based on observations of humans and their interpretations. They are embedded in embodiment, indicated as ‘body and env.’ (where ‘env.’ stands for ‘environment’), generating complex interactions from which behaviour and developmental changes emerge. They are compared with the human's and the generative principles are modified based on the analysis of the comparison. And the whole cycle goes on repetitively. (Online version in colour.)

As the first-order approximation, the dynamical system aspect of continuous autonomous development can be viewed as an ‘autonomous’ dynamical system described by a fixed (time-invariant) differential equation with initial conditions, which are as yet unknown. Observing and analysing the temporal development of the system is one way to infer the governing equation. This may correspond to developmental psychology. Then the obtained hypothesis should be tested by running the hypothetical system and observing the result. In more general terms, we should hypothesize the minimum set of generative principles referring to developmental sciences, embed them in embodiment, let complex interactions take place, observe how the system develops and compare it with the target, i.e. human development, modify the starting set to reduce the observed difference and repeat the cycle (figure 1). This approach, *constructive developmental science*, will provide us with a crucial understanding of the core principles driving the continuous autonomous development.

Since the equation will be high-dimensional and nonlinear, its temporal development will be very complex and can substantially deviate over time on errors and perturbations. This is also a good reason to look at the very beginning of the temporal development, the fetal period, in human development. The uterine environment, although very complex in reality, is still simpler and has fewer external perturbations (stimuli and interactions) compared to the outer world.

### (d) Emergence of sensory–motor interaction

The minimum starting set definitely includes embodiment. Then what is appropriate as the initial driving principle for generating various sensory–motor interactions? Commonly used methods are either predefined (or parametrized) ‘primitive’ actions [30] or random motor signals for ‘motor babbling’ [31]. However, the former will generate only fixed or very limited interactions [39] and the latter will be very inefficient owing to the vast variety of motor patterns to be explored [40]. And neither one has the function of adapting to the dynamics of embodiment. What is desired is a single simplest mechanism/principle that has both explorative and adaptive functionality. A promising candidate, *embodiment-coupled chaotic maps*, is presented in the next section.

### (e) Somatosensory-guided early development to self–other cognition (a hypothesis)

Human behaviour and cognition start from the fetal period. Initially, the muscles are driven by the spinal circuit, in the way discussed above, evoking proprioceptive and tactile signals that are the fundamental sensory modality for embodied behaviour and cognition [41,42]. Continuous autonomous development from such sensory–motor experiences to the cognition of self [43] and other will be the minimum requirement for fusing autonomy and sociality. Saegusa *et al*. [44] provide one such scenario with robotic experiments. In the following, we present a more detailed scenario with an emphasis on realistic human fetal/infant embodiment.

1. Initial prenatal environment: Simple and continuous sensory–motor correlation is provided by the amniotic fluid's resistance to moving body parts. Simple synaptic plasticity, i.e. Hebbian learning or STDP (spike-timing-dependent plasticity) can directly capture the correlational structure. Basic body map and motor control are acquired. Our model and experiments on this phase will be presented in §3.
2. Middle to term prenatal environment: As the fetal body grows, less free space is available in the uterus. The body movements are constrained by the uterine wall. The limbs frequently touch the wall, body and umbilical cord. When a limb swings to hit something, weak and continuous somatosensory signals owing to the fluid resistance are followed by a short blunt peak on hitting flesh. Learning this particular temporal structure can be done using the circuit learned in phase 1 above, and may serve as a basis for handling causality and prediction.
  The self-touch provides a special type of sensory patterns called ‘double-touch’: synchronized tactile signals from two different body parts. This can be learned in a neural layer receiving inputs from the previously established body map. Associated with motor signals, self-touch behaviour can be enhanced, leading to touch exploration of the fetus' own body. Integration of these gives rise to a body schema [45], a sensory–motor representation of self. And non-self objects (i.e. the uterine wall and umbilical cord) are assigned separate sensory–motor representations that become the basis for various object representations.
3. Postnatal environment: Drastically different sensory–motor patterns are provided. When a limb moves to hit something, no tactile signals are received while the limb is moving in the air, followed by an instantaneous peak on hitting an object, quite sharp for rigid ones. Treating this as a unified temporal pattern is rather difficult because temporally distant events have to be correlated. However, the proprioception (and vision) has a partially similar temporal structure to before (i.e. continuous during the movement). And it may facilitate the tactile correlation. Integration of tactile, proprioception, motor and also visual signals at another layer may lead to learning a unified representation of the event and reaching behaviour [46].
  More importantly, touching by other humans constitutes completely novel experiences. This provides tactile stimuli that are non-contingent with self-motor/proprioception signals. If the previous step 2 of sensory–motor learning is complete with predictive functionality, forming the basis of ‘sense of agency’ [47], these novel stimuli present significant prediction errors. This evokes learning [48,49] in the neural layer sending out the predictive signals from efference copy (of motor signals), which requires formation of a new circuit that feeds ‘phantom’ motor signals to the predictive circuit to cancel the errors. This may trigger the self–other distinction with regard to the switching of the input between efference copy and phantom motor signals, and a more complete sense of agency.
  The new circuit has to emulate the motor signals of the target person. It may be created first by ‘free-riding’ a part of the self-motor control/monitoring system because many of the necessary functions are there. This may be the mirror neuron system [50] that treats self and others' action as identical, exhibiting primitive forms of motor imitation. Because it is crucial for achieving the goals of actions to discriminate the signals and models of the self and other, sense of agency is integrated with the models and goal representation, and further enhanced to form self-awareness.
  Eventually, the new circuit will be able to infer what the other person sees/feels and why they act in a particular way from observations and the model of self. This is a model of another person's mind, aka. theory of mind (ToM, [51]), which plays a core role in social cognition.

## 2. Emergence of embodied behaviour

As a single simplest mechanism that has both explorative and adaptive functionality, we proposed and have been investigating *embodiment-coupled chaotic maps*.

**Figure 2.**
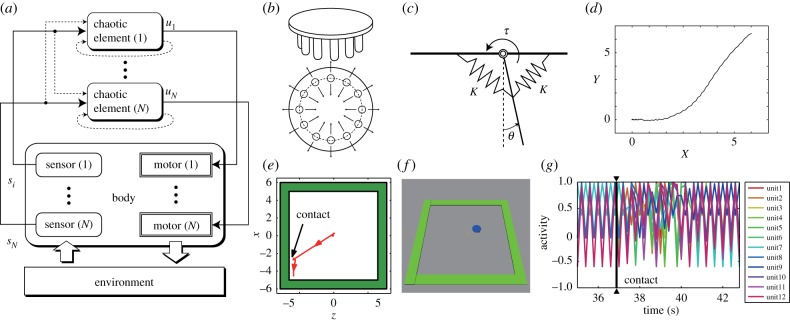
Robot behaviour generation by chaotic maps in sensory–motor loops coupled via embodiment. (*a*) Sensory signals *s_i_* are fed to chaotic elements whose output *u_i_* are fed to motors. In addition, there is weak self feedback and input mixing (broken arrows) (reprinted from [52], Fig. 1, with permission). (*b*) The ‘insect’ type robot with a symmetric structure. It has 12 legs, that can move in radial directions. (*c*) Each leg is suspended by the springs with elastic constant *K*. The angle *θ* is fed as a sensor signal to the corresponding chaos map and its output is linearly mapped to torque *τ* driving the leg (reprinted from [52], Fig. 13, with permission). (*d*) An emergent locomotive trajectory, starting from (0, 0), of the centre point of the locomoting insect-type robot on a horizontal plane with *X-* and *Y*-axes measured in metres (reprinted from [52], Fig. 14, with permission). (*e,f*) Adaptive locomotion. (*f*) The insect-type robot is placed on a flat square area surrounded by walls. (*e*) The robot locomoted toward a wall and after hitting it, the robot autonomously changed the direction of locomotion. (*g*) Temporal trace of the outputs of the 12 chaos maps. After the contact (the time indicated with the black triangles) of the robot with the wall, the so-far stable phase relationship collapsed and became chaotic. But after about 2.5 s, a new phase relationship emerged, resulting in a different leg coordination pattern. And the robot autonomously changed its direction of locomotion adapting to the wall (reprinted from [53], Fig. 3, with permission).

In [52], we reported a series of experiments on robots with each actuator (motor) driven by an output of a chaotic map of an input from a sensor embedded in the actuator (figure 2*a*). In this model, a chaotic map is represented by logistic function: *f*(*x*) = 1 − *αx*^2^, which generates a chaotic time series for 2 ≥ *α* ≥ 1.4011… when its output is fed as the input in the next time step and so on, starting from some initial value. The time series is not divergent but aperiodically oscillating. Also, the map has (quasi-)attractors that vary with *α*.

Our base model assumed no explicit signal interconnections among the maps. A limited amount of symmetric internal connections could be mixed in. The maps were effectively coupled together via body dynamics: the outputs of the maps drove the actuators whose effects were integrated into the body dynamics and affected the sensor readouts depending on each sensor location. Since the body physically interacted with the environment, the body–environment interaction was also integrated into the body dynamics and affect the coupling. The model was inspired by the work by Kaneko & Tsuda [54] on coupled chaotic maps that exhibited various (partially) ordered states. Our model introduced embodiment as the coupling field that was temporally varying and nonlinear.

The robot (see figure 2*b,c*, for example) exhibited various emergent behaviour patterns such as legged locomotion (figure 2*d*) and even adaptation to an abrupt change in the environment structure such as avoiding a wall (figure 2*e,f*) [53]. The transients from the initial random movement to a regular locomotion and from wall hitting to a regular locomotion in a new direction were completed in just a few seconds (figure 2*g*) [53]. Note that the robots were provided with no explicit descriptions (programmes or pre-training results) of any part of the resulting behaviour. The internal connections are symmetric and do not specify any particular coordination. Therefore, the observed behaviour was emergent. The diversifying property of chaos and entrainment property by the (quasi-)attractors gave rise to spontaneous exploration and self-stabilization of various oscillatory modes of the body–environment system. The exploration and stabilization emerge as the intrinsic properties of the whole embodied dynamics without distinct functional components, learning or rewards.

Recently, Shim & Husbands [55,56] greatly enhanced the generality and effectiveness of the framework by introducing sensory adaptation and dynamic order parameters. Der & Martius [57] proposed an alternative framework using a novel synaptic learning mechanism with very similar aims.

## 3. Modelling fetal development

We have been modelling a human fetus *in utero* undergoing spontaneous sensory–motor interactions and learning [58]. A learning and self-organizing neural network (the brain model) was embedded in a realistic physical model of the human fetal body in the uterus, driven to generate spontaneous movements in a similar way to the insect model presented earlier. In terms of sensory–motor interaction, the intrauterine environment is far less complex than the postnatal extrauterine (outside) environment, making it a comparably more feasible target of modelling and experimenting.

Yamada *et al*. [59] report our current version of the model and initial experiments, summarized in the following.

### (a) Embodiment

**Figure 3.**
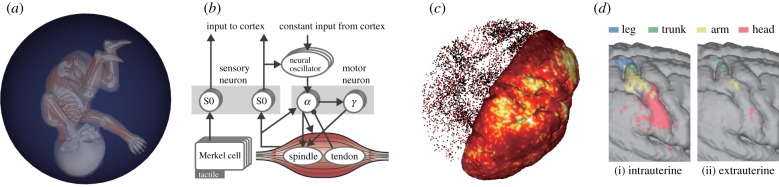
(*a*) The musculo-skeletal model of a human fetus placed in a spherical uterus model filled with amniotic fluid (reprinted from [59], Fig. 1a, with permission). (*b*) The spinal circuit model (reprinted from [59], Fig. 1g, with permission). (*c*) The cortex model with resting state neuronal activity. On the right half, the average firing rate is indicated with colour. On the left, a snapshot of the spikes of excitatory and inhibitory neurons are indicated by red and black dots, respectively (reprinted from [59], Fig. 2a, with permission). (*d*) The responses of the somatosensory area of the cortical model to stimuli on individual body parts after (i) intrauterine and (ii) extrauterine learning (reprinted from [59], Fig. 4b, with permission).

A musculo-skeletal model of an average human fetus at 32 weeks of gestation is shown in figure 3*a*. It has 21 rigid bodies, 20 joints, 390 muscles with embedded proprioceptive receptors (spindles and Golgi tendon organs) and skin with 3000 tactile mechanoreceptors (Merkel cells). It was based on multiple sources of anatomical data including magnetic resonance imaging (MRI) data of historical specimens, CT scan data of skeleton replica and experimental data [60] on the characteristics of the muscles and receptors. The accuracy of the model is particularly important as it defines the embodiment. The fetus body model was placed in a uterus model, an elastic damping membrane sphere filled with simulated amniotic fluid providing buoyancy and resistance to movements. In some of the experiments, the vision was simulated using simple camera models placed in the head providing 16×16 pixel images for left/right fields of view.

The model was placed in a physical dynamics simulator (Open Dynamics Engine [61]). When signals were given to the muscles, they exerted forces according to the muscle dynamics model, driving the skeleton to generate bodily movements and giving rise to receptor responses such as muscle contraction/elongation and fluid resistance on the skin.

### (b) Spinal circuit

A minimum model of the spinal circuit consisting of *α* and *γ* motor neurons, sensory interneurons and neural oscillators was constructed (figure 3*b*). A neural oscillator is a nonlinear oscillator represented with Bonhoeffer–van der Pol (BVP, or FitzHugh–Nagumo) equation. It is widely used to model the central pattern generator (CPG [62]) in the spine and/or brainstem of vertebrates generating regularly repetitive movements such as locomotion, swimming and respiration. It is also known that a coupled nonlinear oscillator system exhibits chaotic behaviour in a range of conditions [63,64]. And we confirmed that the model can be used as a biologically realistic replacement [65] of the chaotic map presented in the previous section. An important point is that unlike other work using CPGs, we deliberately avoided any cross-muscle interconnections. Each muscle was driven by a corresponding neural oscillator and the receptors embedded in the muscle fed back signals to the neural oscillator. The sensory–motor dynamics of multiple muscle-circuits were coupled via embodiment in the same way as the chaotic map-driven robots in the previous section.

When the spinal circuit model drove the musculo-skeletal model, a variety of spontaneous bodily movements, or *emergences of embodied behaviour*, were observed ([59], video). The validity of the resulting movements (limb trajectories) was confirmed by comparing multiple features with those of the human fetus in literature, in terms of Lyapunov exponents, fractal property (long-range correlation), phase-synchronization indices, participating body parts and their movement directions.

### (c) Cortical model

Signals from sensory interneurons in the spinal circuit were fed to the model of the cerebral cortex (figure 3*c*). Leaky integrate and fire (LIF) spiking neuron model with STDP, a standard model of a biological neuron, was used. A network of 2.6 million neurons, the number of excitatory neurons being five times that of inhibitory, with 5.3 billion synaptic connections was placed on the surface (grey matter with undulations) of a three-dimensional model of cerebral cortex with random local connection and cortical connectivity based on MRI and diffusion tensor imaging (DTI) data of 15 preterm human neonates. This version had no grey matter layer structure, area-specific characteristics or subcortical structures. The somatosensory and visual signals were fed into corresponding areas of the primary sensory cortex.

The validity of the model was confirmed by comparing multiple statistical and dynamical features of the spontaneous (resting state) activity without inputs, in terms of lognormal firing rate distribution, excitation–inhibition firing rate balance, greater depolarizations of the average membrane potentials relative to the resting potentials, correlations between structural and functional connectivity across cortical regions and responsiveness to single spikes. In some of the earlier experiments, we adopted a different, simpler cortical model that will be described in §3f.

### (d) Experiment on body map acquisition

The integrated model was run for 1000 s in each learning session. After each session, the cortical responses were examined by stimulating all body parts and their combinations. For comparison, an identical fetus model was placed on a flat plane, simulating an extreme version of preterm birth, for the same experimental procedure. We obtained clear results in which the cortex learned in the normal condition exhibited strong and well segregated (body-part wise) responses, which contrasted with much weaker unsegregated responses in the ‘preterm’ (extrauterine learning) version (figure 3*d*).

This contrast was clearly owing to the different structuring of sensory information. When the fetus moved slowly in the fluid, the movement of each body part took place often independently, resulting in lower somatosensory correlations between the body parts, and the continuous fluid resistance on the moving limbs entailed higher correlation within each of them. On the other hand, when moving in the air, the continuous tactile correlation owing to fluid resistance disappeared. And when moving on the flat plane, a movement of one body part often affects the other, such as when a leg pushes against the ground plane, the ground reaction force on the back of the trunk changes. In summary, the modularity and stability of the correlational structure of the somatosensory signals are higher in the uterus, allowing the cortex to learn a clearly segregated body representation. This fills in the first step of the hypothetical developmental scenario presented earlier.

### (e) Experiment on multi-sensor integration

Multi-modal sensory integration is an important step in early cognitive development. Developmental studies suggest a possibility that somatosensory learning during the fetal period is an important prerequisite for achieving integration in the postnatal period [66]. In order to test this hypothesis, we examined visual–somatosensory multi-modal responses in our embodied brain models.

After learning under the two conditions in the same way as the first experiment, the cortical models were transplanted on to identical bodies whose parameters were set to 40 weeks' gestational age and laid on the flat plane. This was to simulate neonates, one of which underwent intrauterine learning and another, extrauterine. A multi-modal sensory input was generated from the arm movement in front of the eyes. The synchronized visual, proprioceptive and tactile signals were fed to the cortical models and the responses were examined. A significantly stronger multi-modal response was observed in the cortex that learned in the uterus. This supports the hypothesis that intrauterine learning facilitates postnatal multi-sensory integration, which serves as the basis for further cognitive development.

### (f) Closed-loop experiments

In the above experiments, the cortex was passively learning somatosensory/visual inputs. No cortical motor control was assumed because of the immature myelination of the cortico-spinal tract at the target gestational age [67].

However, learning sensory–motor loops is essential for continuous development. In the uterus, the nervous system of a fetus first learns the sensory–motor patterns arising from its spine-driven movements. It then starts to induce its output, modifying the movement patterns and generating new sensory–motor patterns for further learning. This can be the basic process contributing to the motor development observed with human fetuses.

In order to examine such a process, closed-loop experiments were carried out with an earlier version of our fetus model [68]. In this version, the body model was much simpler and less accurate, and the nervous system adopted a continuous non-spiking neuron model. Full connections (many to many) from the cutaneous tactile receptors to the *α* motor neurons and neural oscillators were introduced with Hebbian learning as a coarse generic model of spinal learning. We investigated whether the following well-known events observed with human fetal motor development [69] emerge in our sensory–motor learning model without explicit programming/training: (1) increase in ‘jerky’ (with high acceleration–deceleration) limb movements, (2) increase in hand–face contacts, (3) the two events occur in this order. The experimental results were summarized as follows.

1. Significant increase in jerky limb movements was observed. Comparing the model with a human-like tactile receptor distribution, i.e. dense on face/hands/feet but coarse elsewhere, with a non-human distribution (uniform), the former exhibited significantly more increase. This was because the higher tactile density on the hand gives rise to much stronger tactile–muscle correlation during arm movement than the uniform case, resulting in a stronger positive feedback loop.
2. Significant increase in hand–face contacts was observed. The human-like tactile distribution case exhibited significantly more increase compared to the non-human distribution case. Again, this was because the former case provided a very strong correlation between the high-density tactile sensation from both the hand and face and the muscle activation pattern for the hand–face contact.
3. Event 1 consistently preceded event 2 throughout the systematic change (tripled) of connection gains from the tactile receptors to *α* motor neurons.

The above results suggest (1) the embodied sensory–motor learning loop can reproduce some of the early fetal movement characteristics; and (2) it also partially accounts for the emergence of the global developmental order. The reason for the emergence of such order would be the following: (1) the hands often came near the face in the natural fetal posture imposed by the muscle arrangement and their natural lengths, and (2) the increase in jerky limb movements increased the possibility of the hands actually hitting the face. It is important to note that our model reproduced the development of at least two distinct behaviour patterns continuously on a single system without explicit mechanisms or conditions specific to each. As discussed before, this is a necessary condition for a valid model of development.

## 4. Further issues toward human-like cognition

The above model fills in the very beginning part, with a precise human-like embodiment, of the entire developmental scenario constructed by developmental robotics discussed earlier. So far, our discussions have been focused on sensory–motor interactions. However, there are also other very important factors, such as motivation, emotion, interoception, autonomic nervous system and genetic factors.

Intrinsic motivation (IM), which is generated within an agent and independently of external rewards, is typically defined as a tendency to prefer exploratory actions that have outcomes that are neither too predictable nor too unpredictable [70], with a predictive power that is increased by learning the outcomes. Experiments showed that a system endowed with IM can exhibit continuous development through different cognitive stages [71], suggesting it as an important core mechanism for driving continuous autonomous development [35].

Emotion is particularly important in the context of our discussion because it provides the basis of the value system, an essential component of sociality. Here again, the similar developmental approach to that discussed earlier would be necessary to reveal the principles for general and robust systems. The emotional system is grounded on interoception [49] of internal organs and the autonomic nervous system that controls them [72]. Although there have been numerous studies on modelling human emotion for AI/robots, most of them do not address such deep structures. A few exceptions are WAMOEBA project [73], which constructed robotic emotion based on a self-preservation function defined in terms of battery-level, heat, etc. and *internal robotics* [74], which presented evolutional models of basic drives such as hunger/thirst, pain, illness [74] and *emotional circuit* [75]. But neither accounted for continuous development in detailed human embodiment.

It naturally follows from our earlier discussion that we should start from a fetus model with internal organs, autonomic nervous system and more detailed subcortical circuits, exploring emergence and development of emotion, which is an extremely challenging and cumbersome endeavour. If it is realized, it can be a testbed for the comprehensively integrated view [76] of early human development toward social cognition, which integrates the autonomic nervous system, interoception, sleep, motor control, exteroception, emotional system and others into a hypothetical model of the developmental process from the early fetal period to emergence of social functions such as empathy, and how they may be disrupted resulting in autism spectrum disorder (ASD).

Another important issue is the emergence of human qualities such as morals. A recent work [77] showed that even preverbal human infants (6 and 10 months) exhibited a sense of morality. This suggests a possibility of the emergence of human morality being partially grounded on early sensory–motor and emotional development. If such a process can be modelled, it can suggest how to embed morality and humanity as generative principles deep at the base of an intelligent system, not as a superficial rule-based or data-based mechanism with limitations discussed earlier. Together with the approaches discussed before, this will provide a crucial means to realize human-centred AI/robots.

Needless to say, genetic factors play crucial roles in human development. Vast amounts of knowledge on cognition-related genes have been accumulated. Some of the genetic constraints are already reflected indirectly as structures and parameter settings of the body and nervous system of our model. Precise modelling of genetic effects will require a comprehensive gene network model, possibly extended from an early work [78], and a molecular-level physiological model to be integrated into our current model. Before that, abstracted causal rules on environmental or activity-dependent effects on the body and nervous system can be incorporated. Testing their effects on the embodied interacting/developing model may provide new insights about the impact of the target genes in the context of behaviour and development.

## Data accessibility

This article has no additional data.
